# Spondylocostal dysplasia and brachydactyly associated with TBX6 and IHH variants: A case report

**DOI:** 10.1002/ccr3.6000

**Published:** 2022-07-11

**Authors:** Surasak Puvabanditsin, Michelle Gorbonosov, Kristin Blackledge, Jeffrey Manzano, Matthew Federici, Rajeev Mehta

**Affiliations:** ^1^ Department of Pediatrics Rutgers RWJ Medical School New Brunswick New Jersey USA; ^2^ Department of Family Medicine Rutgers RWJ Medical School New Brunswick New Jersey USA; ^3^ Rutgers Robert Wood Johnson Medical School New Brunswick New Jersey USA

**Keywords:** congenital anomaly, costovertebral malformation, Jarcho–Levin syndrome, spondylocostal dysplasia

## Abstract

We report a preterm male neonate presenting with a short trunk, short neck, low hairline, deformed ears, preauricular skin tag, penoscrotal transposition (PT), palmar crease, short and broad fingers and toes (brachydactyly), hypoplastic and deep‐set nails, metatarsal abductus, and cross‐fused, small echogenic kidneys. Radiologic findings and genetic studies are consistent with spondylocostal dysostosis (SCD) and autosomal dominant brachydactyly. This is the first case report of spondylocostal dysostosis and brachydactyly associated with *TBX6* and *IHH* variants. We reviewed the literature and compared our patient's phenotype with previously reported cases of SCD.

## Graphical abstract

We present a preterm neonate with spondylocostal dysplasia and brachydactyly. The genetic studies identified pathogenic variants (TBX6 and IHH) which correlate with our patient's phenotypes.

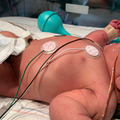

## 1 INTRODUCTION

Costovertebral malformations are rare and result in significant disabilities because they are associated with spinal deformities (e.g., scoliosis, kyphosis) and rib abnormalities that could compromise respiratory function. Spondylocostal dysostosis (dysplasia) [SCD] [OMIM 12600] is an autosomal recessive disorder that is characterized by multiple segmental defects of the vertebrae and abnormalities of the ribs. The phenotypes include a short trunk, short neck, and scoliosis.^1^, ^2^ In neonates, SCD can cause respiratory problems due to reduced size of the thorax. With advancement in diagnosing genetic anomalies, the genetic etiology of congenital vertebral malformation (CVM) is acceptably based on the DNA analysis of single nucleotide polymorphism (SNP) arrays or the next‐generation sequencing technology [whole exome sequence (WES) and whole genome sequence (WGS)].^2^, ^3^, ^4^ We report a new case of SCD with an accompanying genetic analysis.

## 2 CASE PREPORT

**FIGURE 1:**
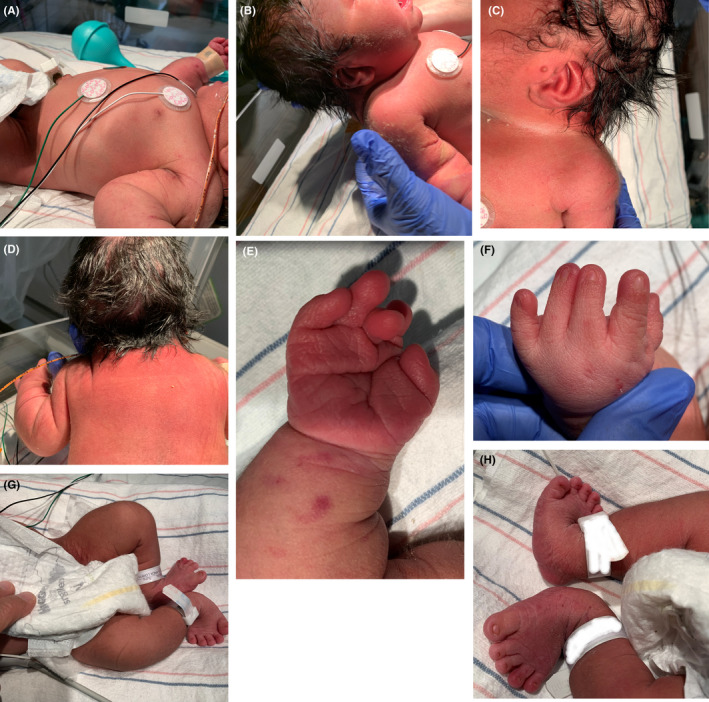
(A and B) Note a body view showing a short trunk and a short neck, (C and D) Note a short neck and a deformed ear (notched helix and prominent antihelix). (E and F) Note a palmar crease and short fingers/nails. (G and H) Note deformed lower extremities with short and broad toes, small nails, and metatarsus abductus

**FIGURE 2:**
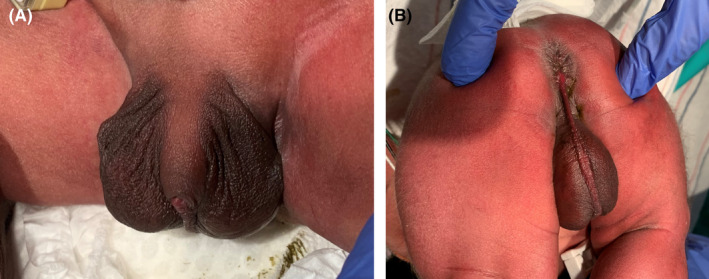
(A and B) Note transposition of the penis and prominent median raphe

**FIGURE 3:**
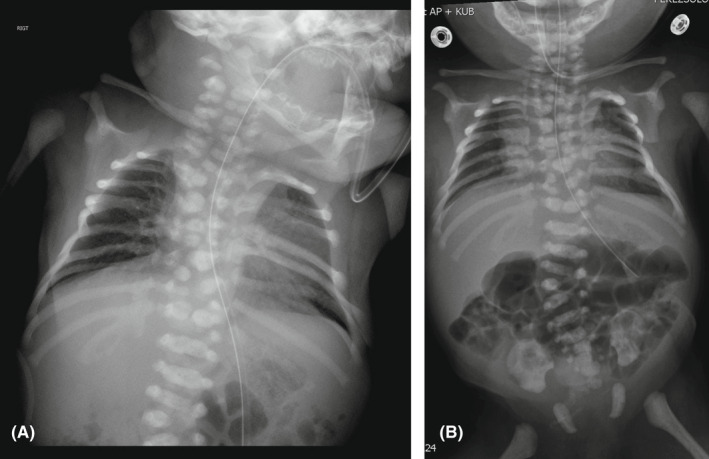
(A and B) Spinal radiographs shows segmentation anomalies of thoracic spines and rib abnormalities, L3 and L4 hemivertebra with lumbar scoliosis

**FIGURE 4:**
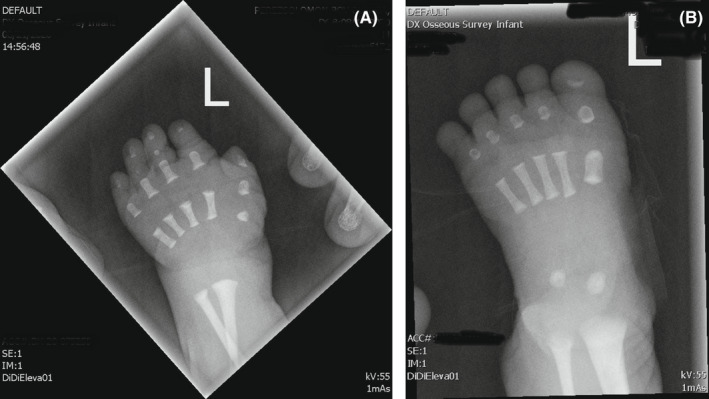
(A) Radiograph of the hand shows absence/short middle phalanges of fingers. (B) Radiograph of the foot shows absence/short middle phalanges of toes

A 2950‐g Hispanic male neonate was born at 36 weeks gestation to a 16‐year‐old primigravida by vacuum‐assisted vaginal delivery. Apgar scores were 9 and 9 at 1 and 5 min, respectively. Pregnancy was uncomplicated. The family history was negative for congenital anomalies, and there was no history of in‐utero exposure to any known teratogens. There was no history of consanguinity. Physical examination revealed a weight of 2950 grams (50th centile), length of 45 cm (5th centile), and head circumference of 34 cm (25th centile). Anomalies noted at birth included the following: cleft soft palate, coloboma of the iris, wide anterior fontanelle, wide sagittal suture, low set ears, short neck, deformed ear, short fingers and toes, transposition of the penis, and prominent median raphe of the scrotum and perineum (Figures 1 and 2). Cranial ultrasound and echocardiography were normal. Chest and spinal radiographs showed segmentation anomalies of the thoracic spines; [“butterfly”] hypoplastic and hemivertebrae were present from the 1st to 12th thoracic vertebral bodies. The ribs were abnormal (fused and dysplastic). L3 and L4 hemivertebra and lumbar scoliosis were also noted (Figure 3). Renal sonography and MRI showed cross‐fused and dysplastic kidneys, and left hydronephrosis. Radiographs of the hands and feet showed hypoplasia/absence of the middle phalanges (Figure 4). Abdominal ultrasound and MRI studies revealed a small enteric duplication cyst (1.6 × 1.5 × 1.3 cm) anterior to the rectum. An upper gastrointestinal contrast study performed at 3 weeks of age because of feeding difficulties (initially thought to be due to the cleft soft palate and disorganized sucking) showed malrotation of the small intestine. The patient underwent an exploratory laparotomy, lysis of Ladd's band, and appendectomy on the 30th day of life. Concurrently, gastrostomy tube placement and Nissen fundoplication were done to help with the feeding. The infant remained in the hospital for 50 days because of the feeding difficulties.

## 3 CYTOGENETIC AND MOLECULAR STUDIES

The karyotype was 46 XY. The whole genome SNP (Single Nucleotide Polymorphisms) microarray analysis was normal. No significant DNA copy number changes in the 2.695 million region‐specific SNPs were detected. The SNP microarray analysis was performed using CytoScan HD platform, which uses over 730,000 SNP probes and 1,953,000 NPCN probes with median spacing of 0.88 kilobase.

A high density of short runs (1.8 Megabase [Mb]) of allele homozygosity (ROH) were observed throughout the genome, consistent with a limited gene pool present in isolated populations. This finding reflects an increased risk of recessive allele pairing. The study also showed additional longer ROH on chromosomes 2, 5, and 16, consistent with a distantly related parental relationship (ROH Bp linear position: chr2:56149474–66,950,851; chr5:17410451–38,717,348; chr16:25974475–35,220,544. Total 41 Mb [1.5% of autosomal genome]).

A sequence analysis and deletion/duplication testing of 109 genes (Invitae Skeletal Dysplasia Panel) were performed by Invitae Laboratory Corporation, San Francisco, California 94103, USA. The result identified four significant variants that possibly related to the clinical findings in our patient (Table 1).

**TABLE 1:** DNA sequencing analysis

Gene	Variant	Zygosity	Associated phenotypes
DVL1	c.1379>G (p.Asn460Ser)	heterozygous	Robinow syndrome
IHH	c.1222G>A (p.Gly408Arg)	heterozygous	AD brachydactyly and AR acrocapitofemoral dysplasia
SGSH	c.67C>T (p.Arg23Trp)	heterozygous	AR mucopolysccharidosis type IIIA (MSP IIIA) [Sanfilippo syndrome]
TBX6	c.699G>C (p.Trp233Cys)	homozygous	Spondylocostal dysostosis and congenital anomalies of the kidney and urinary tract (CAKUT)

Abbreviations: AD, Autosomal Dominant; AR, Autosomal Recessive.

## 4 DISCUSSION

Jarcho–Levin syndrome (JLS) is a congenital vertebral malformation characterized by vertebral body and rib malformations. JLS was first described by Saul Jarcho [a pathologist] and Paul Levin [a neurologist] from Baltimore, USA in 1938.^1^, ^5^ The syndrome represents a hereditary malformation spectrum of short trunk and moderate rib cage restrictions with variable involvement of vertebrae [fused, hemi, and block vertebrae] and ribs [absent to overgrown ribs]. Subsequently, these findings have been termed and known as spondylocostal dysostosis/dysplasia (SCD). The affected individual ethnicity was colored [Anglo‐Saxon]. The DLL3 gene has been linked to SCD.^1^

Lavy–Moseley syndrome was first reported by Norman Lavy from Indiana university, Indianapolis and John Moseley from Mt Sinai, New York City.^1^, ^3^, ^6^ In 1966, Lavy reported infants of the same family from Puerto Rico (PR) who died from severe respiratory insufficiency during infancy. All had small chests, short trunks, symmetric posterior fusion of the ribs with a fan‐like [crab‐like] appearance, and fusion of the occiput of the skull to the first cervical vertebra. Three years later, Moseley reported two more cases of patients with similar findings and used the term spondylothoracic dysplasia/dysostosis (STD) to describe these congenital anomalies.^6^ *MESP2* has been implicated in STD individuals. STD is now described as a rare, pleiotropic genetic disorder with an autosomal recessive inheritance. It is most reported in patients from PR or of documented PR ancestry.^3^ The *MESP2* gene has been implicated in individuals with STD.

Notably, *MESP2* has been implicated in STD individuals. Although the trunk is short in both STD and SCD, the severity of vertebral malformation and clinical course is much worse in STD.

For more than 50 years after the first JLS was reported, there was still confusion with regards to patients with vertebral anomalies and thoracic cage deformities. The term JLS was inaccurately coined to mean SCD or STD. However, SCD and STD are two distinct entities with distinct skeletal anomalies, ethnic connections, modes of inheritance, and survival. Other associated anomalies with SCD include the following: neural tube defects, Arnold‐Chiari malformation, hydrocephalus, diastematomyelia, renal/urinary tract abnormalities, and hydrourteronephrosis.^4^ The clinical course and vertebral malformations are, also, more severe in patients with STD compared with SCD. Our patient is a Caucasian male of Hispanic ethnicity who presented with malrotation of the small bowel, which is a newly reported malformation associated with SCD.

Autosomal recessive variation of *TBX6* has been reported to be associated with segmental defects of the vertebra (SDV), ranging from congenital scoliosis to SCD.^7^, ^8^, ^9^, ^10^ The *TBX6* gene is located on chromosome 16 [16p12.2], is 6,095 bp in size, and contains 8 exons.^8^ Our case supports that the autosomal recessive *TBX6* variant is associated with SCD. *TBX6*, a T‐box gene, encodes a transcription factor which plays an important role in embryonic development of somite (somitogenesis). It plays a key role in human spine development. Among ten patients, Otomo et al. (2019) discovered five 16p11.2 deletions, one splice‐site variant, and five missense variants. Their in vitro functional experiments for missense variations revealed that the majority of the mutations resulted in aberrant *TBX6* protein localization. They found that TBX6 proteins were mislocalized in presomitic mesoderm cells produced from SCD patient‐derived inducible Pluripotent Stem (iPS) cells. Induced cells had lower TBX6 mRNA expression and that its downstream genes were involved in somite formation. The risk haplotype was found in the opposing allele in all congenital scoliosis (CS) patients with missense variations, but not in a SCD patient with bi‐allelic missense variants. Depending on the severity of the *TBX6* function loss, bi‐allelic loss‐of‐function mutations generate a variety of phenotypes, including CS and SCD.^11^

In our patient, the genomic microarray findings are consistent with autosomal recessive allele pairing as products of distantly related parents. The DNA sequence analysis [Invitae Skeletal Dysplasia Panel] identified variants in *TBX6* and *IHH*, which probably correlate with our patient's distinct phenotypes. Our case revealed that SCD could be associated with brachydactyly of both hands and feet, deformed ears, cleft soft palate, and bilateral colobomas of the irises. These associated abnormalities have not previously been reported with SCD. The malformations of the hands and feet (brachydactyly) due to lack of ossification of the middle and distal phalanges, as seen in our patient, have been described with the *IHH* variant (autosomal dominant brachydactyly type A1(BDA1) (MedGen UID 354673).

Based on DNA analysis of SNP arrays and advanced genomic sequencing technology, we are able to identify the genetic etiology of SCD.

Thoracic insufficiency, which causes decreased lung volume and underdevelopment, is the primary cause of morbidity and mortality in SCD.^12^ Tachypnea, tiredness, and pulmonary infections are all possible side effects. Although prospective studies are scarce, breakthroughs in newborn pulmonary care and surgery may be the reason for the drop in infant mortality. Prenatal diagnosis, treatment of lower respiratory infections, and early chest physiotherapy (CPT) were all beneficial in a Teli et al. study of 13 patients with SCD.^12^ Multiple expansion thoracoplasties with vertical expandable prosthetic titanium rib VEPTR equipment were used to treat thoracic insufficiency that would increase with growth (Synthes). In SCD patients with ordinary academic performance, intelligence is spared.^13^, ^14^

In summary, the diagnosis of SCD is based on clinical findings of SCD in neonate [short trunk/thorax, short neck, scoliosis, and possible breathing difficulties] and the radiographic features [multiple segmentation defects of the vertebra, scoliosis, and rib abnormalities (fusions, missing)]. We report a case of a neonate with *TBX6, DVA1*, and *IHH* variants associated with SCD and new associated phenotypes. We illustrate the use of the whole genome microarray and genomic sequencing analysis to identify the genetic etiology of congenital vertebral malformations.

## AUTHOR CONTRIBUTIONS

Surasak Puvabanditsin involved in manuscript design and writing. Michelle Gorbonosov and Rajeev Mehta involved in data analysis and manuscript revision. Kristin Blackledge and involved in manuscript writing and revision. Jeffrey Manzano and Matthew Federici involved in manuscript revision.

## CONFLICT OF INTEREST

The authors declare no conflict of interest.

## CONSENT

Written informed consent was obtained from the patient to publish this report in accordance with the journal's patient consent policy.

## Data Availability

The data that support the findings of this study are available from the corresponding author upon reasonable request.
